# The Influence of Spatial Distance and Trade-Off Salience on Ethical Decision-Making: An Eye-Tracking Study Based on Embodied Cognition

**DOI:** 10.3390/bs15070911

**Published:** 2025-07-04

**Authors:** Yu Yang, Yirui Li, Qingsong Lin, Xuejun Bai

**Affiliations:** 1 School of Educational Science, Nanyang Normal University, Nanyang 473001, China; 20081092@nynu.edu.cn; 2 School of Chinese Language and Literature, Beijing Normal University, Beijing 100875, China; 202311080053@mail.bnu.edu.cn; 3 Tianjin Key Laboratory of Student Mental Health and Intelligence Assessment, Key Research Base of Humanities and Social Sciences of the Ministry of Education, Academy of Psychology and Behavior, Faculty of Psychology, Tianjin Normal University, Tianjin 300387, China

**Keywords:** target verticality, construal level, trade-off salience, ethical decision-making, eye-tracking

## Abstract

Research based on the theory of embodied cognition has revealed that the vertical position of target information in space influences individuals’ construal level, which in turn affects their ethical decision-making. However, previous studies have shown inconsistent effects of construal level on ethical decision-making, which may be moderated by factors such as the manipulation methods of construal level and the salience of trade-offs. This study examines how manipulating the vertical position (high/low) of target information in space—thereby altering perceived spatial distance—impacts ethical decision-making through the lens of embodied cognition, using eye-tracking technology. Experiment 1 isolated the effect of target verticality, while Experiment 2 introduced trade-off salience as an additional factor. Eye-tracking metrics in Experiment 1 revealed that lower target positions significantly increased late-stage cognitive processing difficulty. Experiment 2 demonstrated an interaction between target position and trade-off salience in ethical decision-making. These findings suggest that spatial positioning influences cognitive processing via construal level, with its effects on ethical decision-making moderated by trade-off cues. In summary, this study reveals the significant influence of trade-off salience as a contextual cue in individuals’ ethical decision-making while also providing an embodied cognition perspective to inform decision behavior in human–computer interaction contexts.

## 1. Introduction

The metaphors “looking up at the stars” and “keeping your feet on the ground” encapsulate a fundamental human dilemma: prioritizing future-oriented abstraction or present-focused concreteness. Beyond temporal meaning, these phrases implicate embodied cognition—specifically, the spatial verticality of visual attention. “Looking up” naturally directs gaze to higher visual fields, while “grounded” postures orient toward lower spatial regions (Van Kerckhove et al., 2015). Such physical orientations may subconsciously encode psychological distance, a core construct in construal level theory (CLT), where a greater distance (e.g., spatial, temporal) prompts abstract (“high-level”) mental representations, whereas proximity fosters concrete (“low-level”) processing (Liberman & Trope, 2008).

### 1.1. Construal Level and Embodied Cognition

CLT posits that psychological distance shapes how individuals represent stimuli: distant objects are encoded abstractly (e.g., “exercise”), while near objects are represented concretely (e.g., “playing basketball”; Trope & Liberman, 2003). Spatial distance, a key CLT dimension, is often operationalized via vertical positioning in embodied cognition research. For instance, participants perceive objects in upper visual fields as more distant and abstract than those in lower fields (Nussinson et al., 2019; 2021). This spatial–vertical metaphor influences self-control: high construal levels promote abstract thinking about long-term goals, reducing impulsive behavior (Fujita, 2008; Carrera et al., 2020). Eye-tracking studies support this link: Maheshwari et al. (2022) found that inducing high construal levels via temporal distance increased eye movement control, reflecting enhanced cognitive regulation.

In summary, previous studies typically manipulated psychological distance to influence individuals’ construal levels, with psychological distance generally encompassing four dimensions: temporal distance, spatial distance, social distance, and hypotheticality (Trope et al., 2007). This manipulation is usually achieved through instructional cues. However, research by Van Kerckhove et al. (2015) found that upward (or downward) movements of the head or eyes can also serve as cues for psychological distance (far or near). This embodiment-based approach to manipulating psychological distance has garnered increasing attention in recent years (Nussinson et al., 2019; 2021).

From a psychological perspective, the theory of embodied cognition posits that the human body and mind operate in tandem and resonate with each other (Peng et al., 2019). It emphasizes that when individuals engage in cognitive processing, physical characteristics beyond the brain also exert significant influences, and cognitive processes or states actively extend into the environment in which individuals are situated (Farina, 2021). In natural environments, spatial distance is correlated with the vertical dimension of space: within a person’s field of vision, distant objects typically appear in the upper part of the visual field, while nearby objects often appear in the lower part. Therefore, by manipulating the vertical position of target information in space, one can alter individuals’ perception of the spatial distance to the target, which in turn affects their level of construal. Specifically, a higher (lower) vertical position leads individuals to perceive a greater (shorter) spatial distance, increasing (decreasing) their psychological distance, thereby making individuals more inclined to adopt a high (low) level of construal for representation. Numerous existing empirical studies have corroborated this perspective (Van Kerckhove et al., 2015; Nussinson et al., 2019; 2021).

### 1.2. Construal Level and Ethical Decision-Making

From a psychological perspective, ethical decision-making refers to a four-stage process in which individuals progress from recognizing a moral issue, leading to subsequent moral judgment, moral intention, and finally moral behavior (Craft, 2013). In early moral psychology research, the dominant frameworks were the social intuitionist model and the dual-process model (Haidt, 2001; Greene et al., 2001). Both models acknowledge that moral judgments are influenced by intuitive responses, but the dual-process theory additionally posits that cognition can dominate utilitarian moral reasoning. However, pure intuitionist models were soon challenged by empirical evidence showing that identical behaviors elicit divergent moral judgments across contexts (Eyal et al., 2008). Concurrently, critics argued that the dual-process model’s simplistic linkage of emotion with deontological judgments and cognition with utilitarian judgments fails to elucidate the actual information-processing mechanisms underlying moral decision-making (Amit & Greene, 2012). In early moral research, the dominant models were the social perception model and the dual-process model, but construal level theory (CLT) can simultaneously provide explanations and supplements for the problems and shortcomings of the above two models. Based on an analysis of information processing, CLT highlights differences in the information that individuals focus on when making deontological judgments versus utilitarian judgments, offering new research perspectives for moral studies. As a result, CLT has been widely applied in numerous morality-related studies in recent years (Hofer et al., 2021; Amaral, 2021).

Moreover, in recent years, a representative theory of moral pluralism has emerged—Moral Foundations Theory (Graham et al., 2013)—which seeks to explain the diversity and relativity of moral judgments. Within this framework, care and fairness constitute the individualizing foundations, representing more fundamental moral values. Research has shown that an abstract mindset, which can be influenced by manipulating construal levels, enhances the valuation of these individualizing foundations, thereby leading individuals to prioritize core principles such as fairness (Alper & Yilmaz, 2020).

In summary, construal level influences individuals’ moral judgments and even their final behavioral decisions, with spatial distance being one of the most commonly used means to manipulate construal level. The proximity of spatial distance significantly affects individuals’ ethical decision-making (Gong & Medin, 2012). Although some articles have elaborated on the relationship between construal level and ethical decision-making, the results of previous studies have been inconsistent. A particularly influential study on moral judgment was conducted by Žeželj and Jokić (2014), which systematically validated the findings of Eyal et al. (2008) and Gong and Medin (2012). The results showed that different methods of manipulating construal level yielded results supporting either Eyal et al. (2008) or Gong and Medin (2012). Specifically, manipulating temporal distance produced no significant effects; when manipulating social distance, Žeželj and Jokić (2014) confirmed Eyal et al.’s (2008) finding that a greater social distance led to a higher perceived wrongness of moral violations; and when directly manipulating construal level, Žeželj and Jokić (2014) aligned with Gong and Medin (2012), showing that priming a high construal level reduced the perceived wrongness of moral violations. Žeželj and Jokić (2014) suggested that such divergent results may primarily stem from differences in experimental manipulations.

Additionally, existing research has identified two contradictory theoretical hypotheses that can, respectively, explain such conflicting results (Liberman & Trope, 1998; Gong & Medin, 2012; Hofer et al., 2021; Amaral, 2021). Amaral and Jiao (2023) synthesized them as the abstraction hypothesis and the desirability hypothesis.

The abstraction hypothesis posits that high-level construal representations are relatively abstract and global, leading individuals to focus more on the core features of the processing object while ignoring contingent and detailed features. In contrast, low-level construal representations are concrete and specific, accompanied by a focus on various details related to specific contexts (Hofer et al., 2021). That is, individuals at higher construal levels tend to make more extreme moral judgments, whereas lower-level construals incorporate contextual details, rendering certain violations more forgivable and virtuous acts more expected. Research on psychological/social distance (e.g., proximal vs. distal) and temporal orientation (future-focused vs. present-focused) consistently supports these findings (Agerström & Björklund, 2009; Eyal et al., 2008). Therefore, the abstraction hypothesis suggests that high-level construal leads individuals to prioritize self-values, thereby enhancing self-control and reducing unethical behavior. Conversely, low-level construal increases individuals’ unethical behavior (Eyal et al., 2008; Zhang et al., 2022).

The desirability hypothesis, however, proposes an opposing prediction. Amaral and Jiao (2023) argued that in ethics, particularly utilitarianism, involves considerations of means and ends, where moral actions are judged based on their outcomes rather than the means employed. People’s responses to ethically challenging situations related to goals should vary with the psychological distance of the situation, which results from changes in the decision-maker’s level of construal. The study by Mullen and Monin (2016) found that when people engage in unethical goal-directed behaviors, they typically do so because these actions enable them to achieve a desirable higher-order goal or end state, even if the specific means used to attain this end state are unethical. Amaral (2021) found that high-level construal increases individuals’ focus on the desirability of behaviors. When individuals predict they will confront potential challenges in the distant future, high-level construal prompts them to prioritize the desirability of goals over their feasibility, leading to more unethical behavior: individuals tend to weigh potential personal benefits (i.e., ends) more than the behaviors required to achieve those outcomes (i.e., means) (Liberman & Trope, 1998). In contrast, low-level construal leads individuals to focus more on behavioral feasibility, thereby reducing unethical behavior.

In summary, both the desirability hypothesis and the abstraction hypothesis are rooted in construal level theory, yet they propose opposing outcomes from distinct theoretical perspectives—and both have garnered support from partial empirical research. The divergence in results may also be jointly attributed to factors such as the methods used to manipulate construal level.

Additionally, Pärnamets et al. (2015) argue that where people fixate their gaze typically reflects and interprets their moment-to-moment thought processes, such that even abstract moral problems can reveal decision-making processes through data on gaze location, duration, and other metrics. Regarding the eye movement control process in text reading, the E-Z Reader model proposed by Rayner (2009) and the comprehensive guide to eye-tracking methods and measures provided by Holmqvist et al. (2011) both serve as valuable references for studying individual visual attention during moral situational cognition. Numerous researchers have employed eye-tracking technology to investigate the impact of construal level on decision-making and the underlying changes in eye movement patterns (Pärnamets et al., 2015; Zhang et al., 2022; Li & Chu, 2024). Mézière et al. (2023) argue that eye movement metrics can effectively reflect the cognitive processes of reading comprehension. However, since reading comprehension itself is not a singular cognitive ability but rather a dynamic process modulated by task demands, the selection of appropriate metrics must be task-dependent, with no universal predictor available. Nevertheless, combining early-stage metrics (e.g., first fixation duration) and late-stage metrics (e.g., regression time) can significantly enhance predictive validity. Mézière et al. (2023) further classify eye movement metrics into global metrics and local metrics. Global metrics, such as mean fixation duration, primarily capture overall reading behavior (Rayner et al., 2006), while local metrics can be subdivided into early-stage and late-stage indicators. Late-stage metrics, in particular, reflect subsequent reading processes such as syntactic integration (Vasishth et al., 2013). In the context of moral decision-making during reading, since the focus is on individuals’ post-comprehension cognitive processing and decision-making, researchers should prioritize global metrics and late-stage local metrics.

In summary, previous studies have confirmed that manipulating the vertical position of a target in space can alter an individual’s psychological distance, thereby influencing their level of construal. However, whether this manipulation also affects moral decision-making remains an open question, which constitutes the first research question of this study:

Research Question 1 (RQ1): Does the vertical position of a target in space influence an individual’s moral decisions?

Furthermore, both the abstraction hypothesis and the desirability hypothesis propose that a low level of construal leads individuals to focus more on concrete, detailed content rather than solely on the core features or outcomes of an action. Consequently, a low construal level may increase cognitive load, particularly during late-stage cognitive processing, which could manifest in changes to eye movement patterns. Thus, the second research question is as follows:

Research Question 2 (RQ2): When a target is positioned lower in space, will individuals exhibit prolonged eye movement metrics (e.g., total fixation duration), particularly in late-stage measures?

### 1.3. The Influence of Trade-Off Salience on Ethical Decision-Making

Trade-off is a concept derived from economics, defined as obtaining something of value by giving up another valuable thing (Deng et al., 2016). In ethical decision-making, individuals may abandon certain moral constraints and choose undesirable behaviors to gain benefits (e.g., taking company pens home). However, the moral domain theory posits that whether individuals perceive an issue as a moral decision or prioritize its social impact or personal preferences may be a critical factor leading to the gap between moral judgment and action. That is, the inconsistency between knowing what is right and acting against it stems from individuals not framing the decision as morally relevant (DeTienne et al., 2021). Once individuals recognize a decision as morally relevant, they generally refrain from active trade-offs because this involves their self-image and social identity as moral beings. Consequently, prior research often provides explicit trade-off information in scenarios to force individuals into varying degrees of trade-off (Amaral, 2021; Guzmán et al., 2022).

However, in real-life situations, there is no explicit trade-off system akin to the clear-cut death toll trade-offs between soldiers and civilians proposed by Guzmán et al. (2022). During decision-making, whether individuals engage in trade-offs and to what extent they do so remains uncertain. Particularly in many real-world moral dilemmas, an individual’s actions may become intertwined with sacred values. In such cases, even when trade-offs could yield greater economic benefits, individuals tend to avoid any potential compromise (Tetlock et al., 2000). Consequently, prior research has often treated trade-offs as an inherent component of moral decision-making, effectively forcing individuals to weigh competing values. However, emerging evidence suggests that moral decision outcomes can also be indirectly influenced by altering the type of information decision-makers rely on—specifically, incidental contextual cues such as the timing or location of a scenario (Amaral & Jiao, 2023). These background cues, termed trade-off salience, shape whether individuals subsequently perceive a situation as a moral decision or an alternative decision-making process, ultimately affecting their choices (Khan et al., 2011; Hiller & Woodall, 2019; Amaral & Jiao, 2023). Specifically, Amaral and Jiao (2023) manipulated TS levels while controlling for temporal distance: in the high TS condition, instructions emphasized “to err is human,” discouraging individuals from linking the scenario to the moral domain and encouraging higher levels of trade-off, and in the low TS condition, instructions highlighted “unethical behavior” and “moral context,” reminding individuals that the subsequent decision belonged to the moral domain and prompting lower levels of trade-off. The results showed that under high TS, ambiguous moral violations led individuals to prioritize the desirability of behavioral tendencies over feasibility when the temporal distance was large, enhancing unethical behavioral inclinations (consistent with the desirability hypothesis). Under low TS, emphasizing moral factors caused high-level abstract thinking induced by large temporal distance to strengthen self-control and reduce unethical tendencies (consistent with the abstraction hypothesis).

Khan et al. (2011) proposed that different levels of construal can alter how consumers weigh the relative advantages and disadvantages in their consumption choices. Affinito et al. (2025) further argued that while traditional views suggest higher construal levels promote ethical behavior, organizational failure cases reveal that remote decision-makers often overlook safety and ethics, contradicting conventional wisdom. However, adopting an ethical framing can significantly reduce unethical behaviors. Low trade-off salience, by providing contextual cues that emphasize ethical framing in the decision-making process, may effectively decrease the likelihood of unethical actions.

In summary, existing studies have demonstrated that trade-off salience interacts with construal level to jointly influence ethical decision-making. Additionally, different manipulations of construal level may also affect individuals’ final moral choices. Thus, the third research question (RQ3) of this study examines how the combined effects of target spatial position and trade-off salience influence ethical decision-making:

Research Question 3 (RQ3): Do the vertical position of a target in space and the level of trade-off salience interact to significantly influence ethical decision-making?

Furthermore, trade-off salience affects the type of information individuals rely on during decision-making, which inevitably alters cognitive processing. However, no prior research has investigated eye movement patterns under the joint influence of target spatial position and trade-off salience. Therefore, the fourth research question (RQ4) explores the following:

Research Question 4 (RQ4): How do trade-off salience and target spatial position jointly influence eye movement patterns during decision-making?

## 2. Experiment 1: The Influence of Spatial Distance on Ethical Decision-Making

The vertical position of target information is essentially a form of spatial distance. As a component of psychological distance, spatial distance can influence individuals’ construal levels, thereby affecting their ethical decision-making. Whether the variation in the vertical position of target information (which alters the perceived spatial distance) is sufficient to influence individuals’ ethical decision-making—beyond its impact on construal level—is the first question addressed in this study. Thus, Hypothesis 1 corresponds to RQ 1:

**H1.**  *Individuals’ moral decision-making varies when the vertical position of the target information changes within the spatial layout.*

Previous studies have demonstrated that variations in construal level can induce changes in individuals’ eye movement processing patterns (Maheshwari et al., 2022; Li & Chu, 2024). Therefore, if the vertical position of target information indeed affects individuals’ construal levels, their eye movement processing patterns must change accordingly. Meanwhile, individuals’ eye movement patterns are also significantly correlated with their ethical decision-making. Many classic methods in decision-making research overly rely on introspection and participants’ subjective reports, making it difficult to access information about attention distribution and cognitive load during the decision-making process. In recent years, eye-tracking technology—a non-invasive decision-process-tracking technique—has offered advantages such as non-intrusiveness, wide applicability, and diverse information collection. It can also provide insights into how key factors (e.g., information presentation location, decision difficulty, decision strategies) influence eye movement patterns, thereby illuminating how these factors shape decision-making (Rahal & Fiedler, 2019). Thus, Hypothesis 2 corresponds to RQ 2.

**H2.**  *When a target is positioned lower (vs. higher) in space, individuals exhibit prolonged (vs. shortened) eye movement metrics such as fixation duration, particularly in late-stage measures like total fixation time.*

Using eye-tracking technology, this study investigates the impact of spatial distance on ethical decision-making by manipulating the vertical position (target position, TP) of target information as high (H) or low (L).

### 2.1. Participants

Following Amaral and Jiao (2023) and Li and Chu (2024), a total of 134 college students (4 males, mean age = 20.41 ± 1.55 years) were recruited. Sample size was calculated using G*Power 3.1 (Faul et al., 2009) with a statistical power (β) of 0.80 and independent-samples *t*-test as the statistical method. The calculated effect size (Cohen’s *d*) was 0.49, reaching a moderate effect size. Participants were alternately assigned to the high (H) group or low (L) group based on their order of participation. Each participant received a CNY 5 compensation upon completing the experiment. All participants were right-handed, had normal or corrected-to-normal vision, and no history of neurological disorders. This study was conducted according to the guidelines of the Declaration of Helsinki and approved by the Institutional Review Board of School of Educational Science, Nanyang Normal University (protocol code 202303E and 28 March 2023). Informed consent was obtained from all subjects involved in the study.

### 2.2. Experimental Apparatus

The Eyelink 1000 Plus eye tracker produced by SR Research (Ottawa, ON, Canada) was used, with a sampling rate of 2000 Hz, a screen resolution of 1280 × 1024 pixels, and a refresh rate of 75 Hz. The distance between participants’ eyes and the screen was set at 70 cm.

### 2.3. Experimental Design

A between-subjects design with a single factor of 2 levels (TP: high or low) was employed.

### 2.4. Experimental Materials and Procedures

The experimental materials used SimSun font size 20. Monocular (left eye) data collection was employed. Before the formal experiment, participants underwent a nine-point calibration with an average error of less than 0.5. The experimental materials were six dilemmatic moral scenarios adapted from Greene et al. (2004) and Amaral and Jiao (2023), including Buying a TV, Footbridge, Environmental Policy, Bomb, Submarine, and Euthanasia (see Appendix A.1), presented in a fixed sequential order. All scenarios were narrated in the neutral third-person perspective. For example, the “Buying a TV” scenario read ”Xiao Qin plans to buy a TV. To get a better price, Xiao Qin can tell the salesperson that the same TV is sold at a cheaper price in another store. In this situation, it is said that salespeople always sell the TV at a lower price without verification. How likely is Xiao Qin to tell the salesperson that the same TV is cheaper in another store?” Participants were asked to rate the question “How likely is Xiao Qin to do this?” on a 9-point scale (1 = Absolutely not, 9 = Absolutely yes). Construal level was manipulated by varying the vertical position of target information in the spatial layout—high construal level condition: target information (scenario text and rating question) was presented at the top of the screen, and low construal level condition: target information was presented at the bottom of the screen.

**Figure 1:**
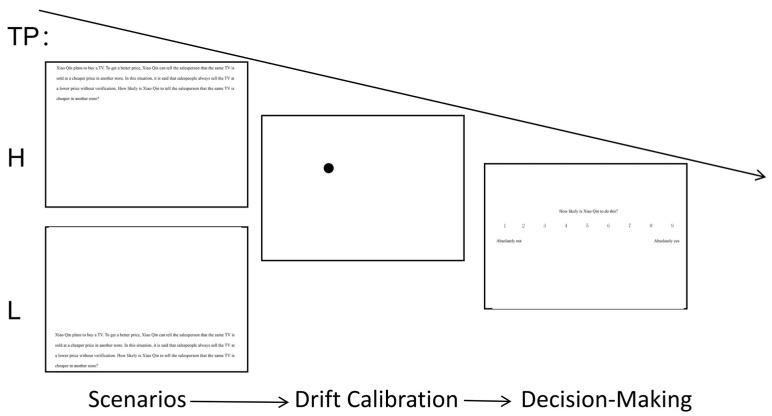
Procedure of Experiment 1.

The task flow is shown in Figure 1. At the beginning of the experiment, participants were instructed to place their left thumb on the spacebar and their right hand on the mouse. Each trial commenced with the presentation of a moral scenario: all text was displayed simultaneously. When TP was high, the first line of the material started from a fixed position in the upper-left corner and followed normal reading habits; when TP was Low, the last line started from a fixed position in the lower-left corner.

Subsequently, pressing the spacebar triggered a drift calibration interface, with the calibration point set at the upper-left corner of the first character in the subsequent decision-making interface (fixed position). After calibration, participants entered the decision-making interface, where they were required to provide a likelihood rating (selected via a mouse) while their eye movement data were recorded. The trial then advanced to the next scenario. The entire experiment lasted approximately 5 min.

### 2.5. Results and Analysis

Experimental data were processed and analyzed using the R software package (4.5.1) and JASP (0.19.1.0).

(1) Ethical Decision-Making Outcomes

The Shapiro–Wilk test indicated that all data were normally distributed (*p* > 0.79), and Levene’s test confirmed the homogeneity of variance between the two groups (*p* = 0.16). Independent-samples *t*-tests revealed no significant difference in ethical decision-making between the two groups (*t*(129.60) = 0.54, *p* = 0.59), with no significant differences across the six scenarios (*p*s > 0.05).

(2) Eye-Tracking Data Outcomes

This study used eye-tracking technology to investigate participants’ cognitive processing and difficulty within the area of interest (AOI), defining the AOI as the central screen region containing scenario texts and rating scales. The study comprised 804 trials in total. Following Maheshwari et al. (2022) and Rayner et al. (2006), trials with missing eye-tracking data or fixation durations shorter than 50 ms in the area of interest (AOI) were excluded (*n* = 17, 2% of total trials), leaving 787 trials for final analysis. Referencing Fiedler and Glöckner (2015), Maheshwari et al. (2022) and Rahal and Fiedler (2019), the following eye-tracking indices were selected: Total Fixation Duration (TFD): the sum of fixation times within the AOI, reflecting late-stage cognitive processing; Fixation Duration Percentage (FDP): the proportion of total fixation time spent on the AOI, indicating attention allocation to the AOI; Fixation Counts (FC): the total number of fixations on the AOI, reflecting cognitive processing load; Fixation Counts Percentage (FCP): the proportion of total fixations occurring within the AOI, indicating processing load in the AOI; Mean Fixation Duration (MFD): the average fixation duration within the AOI, reflecting overall cognitive processing difficulty in the AOI; and First Fixation Duration (FFD): the duration of the first fixation on the AOI, reflecting early-stage processing during reading.

The Shapiro–Wilk test indicated that the data violated the normality assumption; therefore, a logarithmic transformation was applied. Levene’s test confirmed homogeneity (*p*s > 0.55). Using Student’s *t*-tests, the results revealed the following: TFD differed significantly: *t*(785) = 2.78, *p* = 0.006, Cohen’s *d* = 0.20, 95% CI = [−0.13, −0.02]. FDP differed significantly: *t*(785) = 2.77, *p* = 0.006, Cohen’s *d* = 0.20, 95% CI = [−0.08, −0.01]. FC showed no significant difference: *t*(785) = 1.73, *p* = 0.09. FCP showed no significant difference: *t*(785) = 0.94, *p* = 0.35. MFD differed significantly: *t*(785) = 3.58, *p* < 0.001, Cohen’s *d* = 0.26, 95% CI = [−0.05, −0.02]. FFD showed no significant difference: *t*(785) = 1.42, *p* = 0.16. The detailed results are shown in Table 1.

### 2.6. Discussion

An analysis of eye-tracking data revealed significant differences in TFD, FDP, and MFD, indicating that the high spatial position condition led to lower values on late-stage processing indices (TFD and MFD) and relative attention allocation to the area of interest (FDP) compared to the low spatial position condition. No significant differences were observed in early cognitive processing indices (e.g., FFD). This suggests that the low spatial position increased the overall cognitive processing difficulty for the area of interest, with this difficulty primarily manifesting in late-stage cognitive processing, supporting H2.

These results align with both the abstraction hypothesis and the desirability hypothesis. The abstraction hypothesis posits that low construal levels lead to more concrete and deterministic representations, prompting individuals to focus on details related to specific contexts. The desirability hypothesis suggests that low construal levels make individuals more attentive to the feasibility of actions, i.e., the specific behavioral means required to achieve outcomes. Both hypotheses imply that low construal levels induce individuals to process more concrete and detailed information, which may increase late-stage cognitive processing load. Therefore, the changes in participants’ eye movement patterns across high and low target positions indicate that vertical position manipulations effectively altered individuals’ construal levels.

The dissociation between early (FFD) and late (TFD and MFD) processing effects further highlights the dynamic nature of construal-level impacts: spatial position does not affect initial information encoding (reflected in FFD) but significantly influences subsequent deep processing and attention allocation (reflected in TFD, FDP, and MFD). This supports the theoretical framework that construal level primarily modulates post-encoding cognitive operations, such as detail integration and evaluative reasoning.

This lack of significant differences in individuals’ ethical decision-making despite changes in target position may stem from two potential causes: First, the effects generated by position changes based on embodied cognition may be relatively weak. Although such changes influence participants’ construal levels, they are insufficient to impact higher-order ethical decision-making processes. Second, the absence of significant differences in decision outcomes may be attributed to interference from other factors.

## 3. Experiment 2: The Influence of Spatial Distance and Trade-Off Salience Level on Ethical Decision-Making

Numerous previous studies have identified a definitive influence of construal level on moral decision-making, though the results remain inconsistent. This divergence can be explained by the abstraction hypothesis and the desirability hypothesis, respectively. Trade-off salience (TS)—as contextual cues—can effectively shape the type of information individuals rely on during decision-making: enhancing focus on the self-values implicit in actions (thereby reducing tendencies toward unethical behavior) or emphasizing behavioral desirability (thereby increasing unethical tendencies). Therefore, Experiment 2 introduces the factor of trade-off salience level to further explore how spatial distance impacts ethical decision-making after controlling for TS.

This experiment also employed eye-tracking technology. By manipulating the vertical position of target information to influence individuals’ construal levels, and simultaneously using instructions to manipulate the level of trade-off salience (TS) as high (H) or low (L), this study aimed to explore changes in individuals’ ethical decision-making and eye movement patterns. Thus, Hypotheses 3 and 4 correspond to RQ 3, while Hypothesis 5 addresses RQ 4.

**H3.**  *When trade-off salience is high, the target information positioned at a higher spatial vertical location will increase individuals’ tendencies toward unethical behavior.*

**H4.**  *When trade-off salience is low, the target information positioned at a higher spatial vertical location will reduce individuals’ tendencies toward unethical behavior.*

**H5.**  *A high or low level of trade-off salience will affect individuals’ eye movement patterns.*

### 3.1. Participants

Following Amaral and Jiao (2023) and Li and Chu (2024), a total of 112 college students were recruited (4 males, mean age = 20.42 ± 1.66 years), with 111 participants completing the experiment effectively. Using G*Power 3.1 (Faul et al., 2009) to calculate the effect size with a statistical power (β) of 0.80 and a two-way analysis of variance (ANOVA) as the statistical method, the current study obtained an effect size of Cohen’s f = 0.27, approaching a medium effect size. Participants were sequentially assigned to the HH group, HL group, LH group, and LL group according to their order of participation. Each participant received CNY 5 as compensation after completing the experiment. All participants were right-handed, had normal or corrected-to-normal vision, and no history of neurological disorders. This study was conducted according to the guidelines of the Declaration of Helsinki and approved by the Institutional Review Board of School of Educational Science, Nanyang Normal University (protocol code 202303E and 28 March 2023). Informed consent was obtained from all subjects involved in the study.

### 3.2. Experimental Apparatus

The apparatus used is the same as that in Experiment 1.

### 3.3. Experimental Design

A 2 (TP: H, L) × 2 (TS: H, L) between-subjects design was adopted. Twenty-eight participants were recruited for each group.

### 3.4. Experimental Materials and Procedures

The experimental materials comprised nine moral dilemmas adapted from Greene et al. (2004) and Amaral and Jiao (2023), including Forgot Lunch, Choking for Money, The Architect, Standard Trolley, Extra Sweater, Confidential Information, Donation, Crying Baby, and Vaccine Test (see Appendix A.2), presented in a fixed sequential order. Prior to the experiment, instructional prompts for manipulating trade-off salience (TS) levels (Amaral & Jiao, 2023) were presented, with all other procedures identical to Experiment 1.

High Trade-Off Salience (TS-H) Instructions: “*When facing unexpected situations, people often engage in behaviors that may or may not align with their character. Our goal is not to judge anyone but to understand how different individuals respond to such circumstances. Below are scenarios people might encounter in daily life. For each situation, imagine yourself in the described context and indicate what you think you would do. No one is perfect, so regardless of your reaction, many others might act similarly. All responses are completely anonymous, so please be honest about what you believe you would do.*” Low Trade-Off Salience (TS-L) Instructions: “*When people have the opportunity to act unethically, they often behave in ways that contradict their character. Our purpose is to explore how individuals respond when faced with the chance to engage in unethical behavior. Below are several moral dilemmas you might encounter. For each situation, imagine yourself in the described scenario and indicate what you think you would do. All responses are entirely anonymous, so please answer honestly*.”

### 3.5. Pre-Test

To confirm the effectiveness of manipulating trade-off salience (TS) levels, a pre-test was conducted with reference to Amaral and Jiao (2023). Forty-six participants (one male, mean age = 20.98 ± 0.71 years) were recruited from the same participant pool, and these participants did not participate in the formal experiment. They also received CNY 5 as compensation.

**Figure 2:**
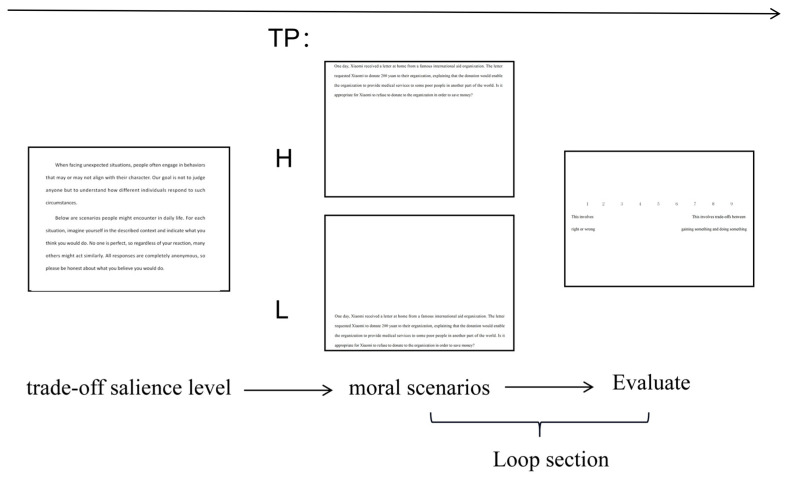
Flowchart of the pre-test.

First, participants were presented with materials manipulating TS levels (high or low) and then entered the moral scenario presentation interface (with the target position set as high or low). Finally, participants were asked to rate the trade-off degree of the nine scenarios in Experiment 2 using a 9-point scale (1 = This involves right or wrong, 9 = This involves trade-offs between gaining something and doing something). The procedure is illustrated in Figure 2 (taking the low trade-off salience instructional prompts as an example).

The Shapiro–Wilk tests indicated that the data violated normality assumptions (all *p*s < 0.05). Using the ARTool package (0.11.2) in R (4.5.1), we conducted Aligned Rank Transform (ART) analyses with TP and TS as between-subjects factors. The results showed that only the main effect of TS was significant, *F*(1, 42) = 29.33, *p* < 0.001, η^2^ = 0.35, 95% CI = [0.16, 1.00]; the main effect of TP was nonsignificant, *F*(1, 42) = 0.528, *p* = 0.47, and the interaction effect was nonsignificant, *F*(1, 42) = 2.46, *p* = 0.12. Further analysis showed that high trade-off salience significantly enhanced participants’ perceived trade-off degree in the scenarios (significant differences were observed in six of the nine scenarios; see Table 2), demonstrating the effectiveness of the trade-off salience manipulation.

### 3.6. Results and Analysis

Experimental data processing and analysis for the results of the remaining six scenarios after excluding three non-significant scenarios were conducted using the R software package (4.5.1) and JASP (0.19.1.0). First, 1 participant in the LH group who failed to complete the experiment due to calibration issues was excluded, and the results of the remaining 111 participants were analyzed.

(1) Ethical Decision-Making Outcomes

**Figure 3:**
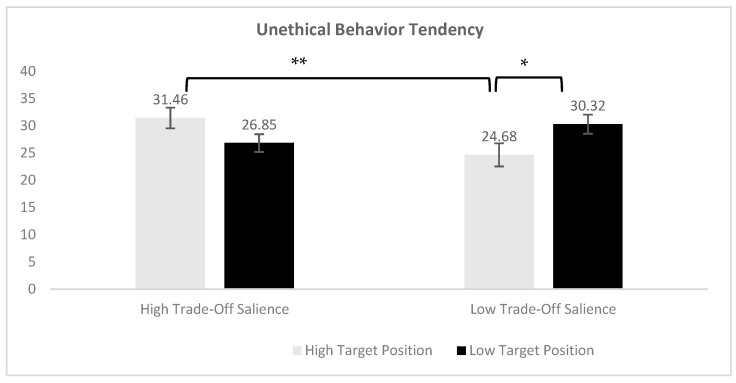
Unethical behavior tendency. Note: * *p* < 0.05, ** *p* < 0.01.

The Shapiro–Wilk test confirmed the normality of the data (*p* = 0.72), and Levene’s test indicated a homogeneity of variance (*p* = 0.65). A two-way analysis of variance (ANOVA) revealed that the main effect of TP was nonsignificant, *F*(1, 107) = 0.12, *p* = 0.73. The main effect of TS was also nonsignificant, *F*(1, 107) = 1.26, *p* = 0.26. However, a significant interaction effect was observed, *F*(1, 107) = 12.06, *p* < 0.001, η^2^ = 0.10. Simple effect analysis showed that the HH group (*M* = 31.46, *SD* = 8.94) had significantly higher tendencies toward unethical behavior than the HL group (*M* = 24.68, *SD* = 7.41), *p_Bonferroni_* = 0.009, 95% CI = [1.20, 12.37]. Additionally, the LL group (*M* = 30.32, *SD* = 6.41) also showed significantly higher tendencies than the HL group, *p_Bonferroni_* = 0.046, 95% CI = [−11.23, −0.05]. These results indicate that individuals exhibited significantly stronger inclinations toward unethical behavior under conditions of high target position with high trade-off salience (HH) and low target position with low trade-off salience (LL) compared to the high target position with low trade-off salience (HL) condition, as illustrated in Figure 3.

(2) Eye-Tracking Data Outcomes

The areas of interest (AOIs) and eye-tracking metrics were identical to those in Experiment 1. The study initially included 666 trials. Following standard exclusion criteria (Maheshwari et al., 2022), we removed trials with missing eye-tracking data and trials with AOI fixation durations < 50 ms. This resulted in the exclusion of 15 trials (2.25%), leaving 651 trials for final analysis. The Shapiro–Wilk test indicated violations of normality (*p* < 0.05), so a logarithmic transformation was applied. Levene’s test confirmed homogeneity (*p*s > 0.55). For TFD, the main effect of TP was not significant, *F*(1, 647) = 0.15, *p_Bonferroni_* = 0.70. The main effect of TS was significant, *F*(1, 647) = 9.23, *p_Bonferroni_* = 0.002, η^2^ = 0.014. Post hoc tests: high TS < low TS, 95% CI [−0.14, −0.03]. The interaction effect was not significant, *F*(1, 647) = 2.74, *p_Bonferroni_* = 0.10. For FDP, the main effect of TP was not significant, *F*(1, 647) = 0.02, *p_Bonferroni_* = 0.89. The main effect of TS was significant, *F*(1, 647) = 17.04, *p_Bonferroni_* < 0.001, η^2^ = 0.03. Post hoc tests: high TS < low TS, 95% CI [−0.11, −0.04]. The interaction effect was not significant, *F*(1, 647) = 0.86, *p_Bonferroni_* = 0.36. For FC: The main effect of TP was not significant, *F*(1, 647) = 0.63, *p_Bonferroni_* = 0.43. The main effect of TS was significant, *F*(1, 647) = 4.91, *p_Bonferroni_* = 0.03, η^2^ = 0.01. Post hoc tests: high TS < low TS, 95% CI [−0.11, −0.01]. The interaction effect was not significant, *F*(1, 647) = 1.30, *p_Bonferroni_* = 0.25. For FCP, the main effect of TP was not significant, *F*(1, 647) = 0.56, *p_Bonferroni_* = 0.45. The main effect of TS was significant, *F*(1, 647) = 18.65, *p_Bonferroni_* < 0.001, η^2^ = 0.03. Post hoc tests: high TS < low TS, 95% CI [−0.08, −0.03]. The interaction effect was not significant, *F*(1, 647) = 0.75, *p_Bonferroni_* = 0.39. For MFD, the main effect of TP was not significant, *F*(1, 647) = 0.68, *p_Bonferroni_* = 0.41. The main effect of TS was marginally significant, *F*(1, 647) = 6.06, *p_Bonferroni_* = 0.01, η^2^ = 0.01. Post hoc tests: high TS < low TS, 95% CI [−0.05, −0.01]. The interaction effect was not significant, *F*(1, 647) = 2.20, *p_Bonferroni_* = 0.14. For FFD, the main effect of TP was not significant, *F*(1, 647) = 0.003, *p_Bonferroni_* = 0.96. The main effect of TS was not significant, *F*(1, 647) = 0.15, *p_Bonferroni_* = 0.70. The interaction effect was not significant, *F*(1, 647) = 0.29, *p_Bonferroni_* = 0.59. The detailed results are shown in Table 3.

### 3.7. Discussion

In terms of the behavioral outcomes of ethical decision-making, when TS was low and TP was high, individuals’ tendency to engage in unethical behavior was significantly lower than when both TS and TP were low, confirming H4 and aligning with the findings of Amaral and Jiao (2023). This indicates that altering the target position not only influences individuals’ construal level but also induces changes in their ethical decision-making through this effect.

Additionally, the construal level factor interacts with trade-off salience (TS) to jointly influence ethical decision-making: When TS is low, emphasizing moral concepts leads individuals to more easily associate subsequent decisions with the moral domain during the decision-making process. In this case, individuals prioritize moral issues over other concerns and make decisions consistent with moral standards. Meanwhile, high construal level, which enhances self-control, further motivates individuals to reduce unethical behavioral tendencies.

The eye-tracking data revealed that when TS was low, participants showed significantly higher values on TFD, FDP, FC, FCP and MFD (indices reflecting cognitive processing difficulty) compared to when TS was high. This indicates that low TS significantly increases individuals’ cognitive processing difficulty relative to high TS. Previous research on trade-off types in decision-making has found that changes in trade-off types affect decision-making difficulty and negative emotions, with trade-offs involving sacred values eliciting stronger negative emotions than those not involving sacred values (Hanselmann & Tanner, 2008). In this experiment, emphasizing moral concepts under low TS necessarily led individuals to engage more in trade-offs related to sacred values, thereby triggering changes in their cognitive processing.

However, the smaller TFD, FDP, and MFD values observed under high TP in Experiment 1 were not replicated in this experiment. Specifically, the eye-tracking results did not show an interaction effect but only significant differences across TS levels. This may be because the effect of individual construal level changes induced by target position manipulation was smaller, while the cognitive processing changes caused by decision-type differences were more pronounced, masking the influence of spatial distance. Numerically, the means of TFD, FDP, and MFD under the HH condition were the smallest across all conditions, a trend consistent with the results of Experiment 1 (although this result was not significant).

Amaral and Jiao (2023) demonstrated the stable influence of TS (trade-off salience) between construal level and ethical decision-making through a series of behavioral experiments. However, it is difficult to explore the underlying mechanisms from the perspective of behavioral results alone. This experiment adopted eye-tracking technology to supplement the behavioral results, demonstrating that TS has a significant impact on individuals’ eye movement patterns (and the underlying cognitive processing). Additionally, it was found that the impact of TS level on eye-tracking indicators was mainly reflected in later-stage indicators such as TFD (total fixation duration).

## 4. Discussion

The study of ethical decision-making is a matter of great importance for the overall well-being of society. The development of moral standards in individuals and society as a whole faces numerous challenges, requiring the collective efforts of policymakers, administrators, and all members of society. However, at present, we still know very little about the process of individual ethical decision-making. Construal level and trade-offs may be significant factors influencing an individual’s ethical decisions.

### 4.1. Key Findings

In both experiments of this study, the vertical position of target information in space was manipulated to explore its effects on ethical decision-making and eye movement patterns. In Experiment 1, a significant effect of target position on eye movement patterns was found: participants in the high target position condition exhibited shorter total fixation duration, a smaller proportion of fixation time on the target area, and shorter average fixation duration. These results indicate that changes in target position indeed influence individuals’ construal levels, consistent with the abstract hypothesis and the desirability hypothesis, which suggest that lower construal levels lead individuals to pay extra attention to concrete detail information. However, participants’ ethical decision-making did not change significantly. Two possible explanations for this exist.

First, although manipulating target information position based on embodied cognition affected participants’ construal levels, this effect was insufficient to influence higher-order ethical decision-making processes. Second, the lack of significant changes in decision outcomes may be attributed to confounding factors.

Therefore, in Experiment 2, the factor of trade-off salience (TS) was added for further investigation. The results revealed a significant interaction between the target information position (TP) and TS level: when TS was low and TP was high, individuals’ tendency toward unethical behavior was significantly lower than when TS was low and TP was low. This interaction effect is largely consistent with the findings of Amaral and Jiao (2023), and studies by Khan et al. (2011) and Affinito et al. (2025) have also demonstrated the possibility of such effects from other perspectives. This indicates that although the influencing factors of construal level were altered, its interaction pattern with TS remained relatively stable.

As a contextual factor affecting decision outcomes, TS may lead participants to attribute different meanings to the current situation:

When TS is low, terms like “unethical” inform participants that the decision falls within the moral domain. At this point, individuals need to engage in a comprehensive evaluation of potential negative consequences (the adverse effects of unethical behavior) and positive personal implications (the beneficial impacts of unethical actions for the individual), leading to repeated trade-offs. Consequently, when TP is high, individuals exhibit greater caution in navigating this trade-off, thereby avoiding tendencies toward unethical behavior.

In contrast, when TS is high, phrases such as “*No one is perfect*, *so regardless of your reaction*, *many others might act similarly.*” blur the connection between the situation and morality, encouraging participants to engage in more extensive trade-offs. At this level, high construal level leads individuals to overlook concrete, low-level details, reducing their perception of moral violations—a finding consistent with Affinito et al. (2025). This, in turn, results in individuals paying less attention to their moral obligations and focusing more on the desirability of the behavior, thereby increasing their tendency toward unethical actions in line with the “desirability hypothesis”.

### 4.2. Implications

This suggests that in the practical design of human–computer interaction, adjusting the vertical positioning of information on the interface can influence individuals’ critical decision-making.

For example, in medical or other high-stakes decision-making systems, placing core ethical issues at the top of the screen can activate healthcare professionals’ high-level construals by leveraging the “high goal position” effect. This primes the moral salience of the scenario, helping them focus on ethical principles.

In financial investment platforms, if the goal is to alert users to short-term profit temptations, designers could employ low-TS framing—such as warning messages like “This action may involve integrity risks.”—to enhance users’ moral sensitivity. Simultaneously, positioning such information in a high-goal location can strengthen self-control while reducing susceptibility to short-term incentives.

Weinmann et al. (2016) also discussed in their review on digital nudging that the environmental design of information presentation can exert subconscious influence on individual choices. Schneider et al. (2018) systematically examined the ethical considerations in digital nudging design, including the following: prioritizing user well-being—avoiding guiding users toward choices that yield short-term benefits but long-term harm (such as inducing overconsumption); the transparency principle—designs should not deliberately conceal information or manipulate cognition, but rather preserve users’ perception of choice autonomy; and personalized ethics—when utilizing user data for nudging, privacy protection standards must be adhered to, avoiding the excessive mining of personal information. In summary, these perspectives demonstrate that the findings of this study can be effectively extended to practical applications in human–computer interaction, while greater attention should be paid to corresponding ethical principles during implementation.

The changes in eye movement patterns further validate the potential role of TS in the relationship between construal level and ethical decision-making: The failure to replicate the TP effect observed in Experiment 1 in Experiment 2’s eye-tracking data may be precisely due to the stronger effect of TS, which masked the influence of TP. Previous research on trade-off paradigms has demonstrated significant dissociations in decision-making difficulty, affective responses, and neural correlates between choices involving sacred values versus those that do not (Hanselmann & Tanner, 2008; Duc et al., 2013). These findings have been consistently replicated across multiple decision-making contexts, including the exploration–exploitation trade-off, value–uncertainty trade-offs (involving decisions between known rewards and uncertain outcomes), and related behavioral paradigms that similarly engage evaluative and cognitive control processes (Aberg et al., 2022; Canessa, 2025). This is consistent with the significant changes in eye movement patterns in Experiment 2 and indicates that TS influences the meaning participants attribute to the situation by providing contextual cues, altering the specific information they rely on during decision-making and leading to significant changes in their behavioral tendencies.

### 4.3. Limitations and Future Research

Trade-offs represent a fundamental challenge in decision-making, where individuals must reconcile competing demands. Traditional research has often framed trade-offs as forced-choice scenarios, requiring an explicit selection between alternatives. However, trade-off salience—operating as contextual information—can implicitly shape attitudes and decisions without coercing participants into a specific choice. Crucially, such contextual cues may not be limited to sacred-value trade-offs but could extend to a wide range of decision-making contexts.

This raises an important empirical question: can subtle contextual cues similarly guide decision outcomes in other types of trade-offs? Furthermore, since participants are not compelled to make a forced selection, the underlying cognitive-affective processes (e.g., emotional responses) and their neural and oculomotor correlates (e.g., eye-tracking and neuroimaging measures) may differ significantly from those observed in constrained decision paradigms. These possibilities warrant further investigation.

Trade-off salience is a newly proposed concept, and its essential definition still requires further research. However, several existing concepts are related to trade-off salience. For example, Bandura (1999) proposed moral disengagement, which operates through eight mechanisms—moral justification, euphemistic labeling, advantageous comparison, the displacement of responsibility, the diffusion of responsibility, the disregard or distortion of consequences, dehumanization, and the attribution of blame—to weaken and suppress an individual’s moral self-regulatory system. This leads to specific cognitive tendencies in behavior, ultimately redefining one’s responsibility for consequences and reducing empathy for the suffering of victims. Similarly, trade-off salience provides an opportunity to justify weakening or suppressing the moral self-regulatory system by emphasizing that *“No one is perfect*, *so regardless of your reaction*, *many others might act similarly.”*, thereby altering ethical decision-making.

The ethical framework is likely a concept more closely associated with trade-off salience. Chen et al. (2020) demonstrated that adopting an ethical framework can reduce unethical behavior. That is, framing behavior from a moral perspective increases ethical conduct or at least decreases unethical actions (Affinito et al., 2025). This is referred to as the “ethical framing effect” (Kern & Chugh, 2009). Trade-off salience influences individuals’ final decisions precisely by reminding them of the ethical implications of their behavior. Research by Kouchaki et al. (2013) also found that when individuals are reminded of the ethical implications of their actions, they are more likely to behave morally.

Furthermore, concepts such as moralization (Rozin, 1999) and moral intensity (Jones, 1991) may also bear certain relationships with trade-off salience. To some extent, these concepts can influence individuals’ cognitive tendencies regarding moral issues by altering the contextual cues of moral situations, thereby prompting individuals to weigh relatively moral versus relatively immoral options in their final decisions, ultimately affecting their ethical decision-making. However, the precise relationships between these concepts and trade-off salience still require further investigation in future research.

## 5. Conclusions

(1) The vertical position of information in space influences eye movement patterns, indicating that target position affects individuals’ construal levels.

(2) Trade-off salience interacts with construal level to jointly influence ethical decision-making.

## Figures and Tables

**Table 1:** Eye-tracking indicators in Experiment 1.

	TP:H		TP:L			
Eye-Tracking Indicators	*M*	*SD*	*M*	*SD*	Welch’s *t*	Cohen’s *d*
TFD	1532.48	1369.37	1843.84	1746.42	2.78 **	0.20
FDP	0.475	0.210	0.521	0.218	2.77 **	0.20
FC	180.84	85.39	190.74	88.47	1.73	
FCP	7.55	6.40	8.38	7.03	0.94	
MFD	0.584	0.175	0.599	0.179	3.58 ***	0.26
FFD	180.84	85.39	190.74	88.47	1.42	

Note: ** *p* < 0.01, *** *p* < 0.001.

**Table 2:** The results of the pilot experiment for trade-off salience manipulation.

Scenarios	*F*	*p*	η^2^	TS	*M*	*SD*
Forgot Lunch	2.42	0.13	0.03	H	5.86	2.23
				L	4.67	2.44
Choking for Money	2.17	0.15	0.04	H	2.32	1.84
				L	1.67	1.37
The Architect	9.69 **	0.003	0.19	H	4.18	1.87
				L	2.54	1.56
Standard Trolley	2.38	0.13	0.04	H	2.46	1.99
				L	1.75	1.51
Extra Sweater	16.36 ***	<0.001	0.24	H	5.14	2.05
				L	2.88	2.11
Confidential Information	9.28 **	0.004	0.18	H	4.46	1.97
				L	2.71	1.81
Donation	6.88 *	0.01	0.13	H	5.32	2.21
				L	3.58	2.34
Crying Baby	9.33 **	0.004	0.18	H	5.64	2.04
				L	3.75	2.11
Vaccine Test	5.10 *	0.03	0.10	H	3.00	1.75
				L	2.04	1.33

Note: * *p* < 0.05, ** *p* < 0.01, *** *p* < 0.001.

**Table 3:** Results of eye-tracking indicators in Experiment 2.

	TP:H		TP:L		
	H	L	H	L	*p_Bonferroni_*
TFD	1162 (977)	1562 (1270)	1197 (890)	1420 (1287)	TS: H < L ***
FDP	0.50 (0.25)	0.59 (0.23)	0.50 (0.24)	0.57 (0.24)	TS: H < L ***
FC	5.62 (4.19)	7.10 (5.60)	5.65 (4.09)	6.29 (5.21)	TS: H < L *
FCP	0.59 (0.20)	0.66 (0.18)	0.59 (0.20)	0.64 (0.19)	TS: H < L ***
MFD	204 (72)	226 (73)	222 (96)	225 (88)	TS: H < L *
FFD	197 (105)	197 (99)	191 (74)	197 (81)	

Note: * *p* < 0.05, *** *p* < 0.001.

## Data Availability

The data that support the findings of this study are available from the corresponding author upon request.

## Abbreviations

The following abbreviations are used in this manuscript:

TP	Target Position
TS	Trade-Off Salience
TFD	Total Fixation Duration
FDP	Fixation Duration Percentage
FC	Fixation Counts
FCP	Fixation Counts Percentage
MFD	Mean Fixation Duration
FFD	First Fixation Duration

## Appendix A

### Appendix A.1

Buying a TV: Xiao Qin plans to buy a TV. To get a better price, Xiao Qin can tell the salesperson that the same TV is sold at a cheaper price in another store. In this situation, it is said that salespeople always sell the TV at a lower price without verification. How likely is Xiao Qin to tell the salesperson that the same TV is cheaper in another store?

买电视：小秦打算购买一台电视机。为了得到更优惠的价格，小秦可以告诉销售人员，另一家商店以更便宜的价格出售同款电视机。在这种情况下，据说销售人员总是不经核实，也会以更低的价格出售电视机。 小秦告诉销售人员同一款电视机在另一家商店售价更便宜的可能性有多大？

Footbridge: A runaway trolley is hurtling down the tracks toward five workers, who will be killed if the trolley continues. Xiao Lin is standing on a footbridge over the tracks, next to a stranger. The only way to save the five workers is to push the stranger off the bridge onto the tracks below, where his large body will stop the trolley. If Xiao Lin does this, the stranger will die, but the five workers will survive. Is it morally appropriate for Xiao Lin to push the stranger onto the tracks to save the five workers?

人行桥：一辆失控的电车正沿着铁轨向五名工人驶去，如果电车继续行驶，他们将被撞死。小林站在铁轨上的一座人行桥上，旁边有一个陌生人。拯救这五个工人生命的唯一办法是把这个陌生人推下桥，推到下面的轨道上，在那里他庞大的身体将停止电车。如果小林这样做，那个陌生人会死，但那五个工人会得救。为了保存那五个工人，小林把陌生人推到铁轨上合适吗？

Environmental Policy: Xiao Dong is a member of a government legislature deciding between two environmental hazard policies: Policy A: 90% chance of no deaths, 10% chance of 1000 deaths. Policy B: 92% chance of no deaths, 8% chance of 10,000 deaths. Is it morally appropriate for Xiao Dong to vote for Policy B over Policy A?

环境政策：小董是政府立法机关的成员。立法机关正在两种不同的环境危害政策之间作出决定。策略A有90%的可能性不造成任何死亡，有10%的可能性造成1000人死亡。政策B有92%的机会不造成死亡，8%的机会造成10,000人死亡。小董投票支持政策B而不是政策A合适吗？

Bomb: Xiao Zhou is negotiating with a powerful and determined terrorist who is about to detonate a bomb in a crowded area. One advantage Xiao Zhou has is that the terrorist’s son is under their custody. The only way to prevent the detonation is for Xiao Zhou to break the terrorist’s son’s arm on camera and threaten to break the other arm if the terrorist does not stand down. If the bomb explodes, it will kill thousands of people. Is it morally appropriate for Xiao Zhou to break the terrorist’s son’s arm to prevent the terrorist from killing thousands?

炸弹：小周正在与一个强大而坚定的恐怖分子谈判。这个恐怖分子即将在一个拥挤的地区引爆炸弹。小周的一个优势是恐怖分子的儿子在他（她）的监护下。小周只能做一件事来阻止恐怖分子引爆炸弹，即在镜头前打断恐怖分子儿子的一只胳膊，然后威胁说如果不放弃引爆炸弹，就打断恐怖分子儿子的另一只胳膊；如果炸弹被引爆，会杀死成千上万的人。为了防止恐怖分子用炸弹杀死成千上万的人，小周打断恐怖分子儿子的胳膊合适吗？

Submarine: Xiao Wu is the captain of a military submarine. An explosion on board has destroyed most of the oxygen supply and injured a crew member, who will die from their wounds regardless of treatment. The remaining oxygen is insufficient for all crew members to surface safely. The only way to save the other crew members is for Xiao Wu to shoot the injured crew member, preserving enough oxygen for the rest to survive. Is it morally appropriate for Xiao Wu to kill the fatally injured crew member to save the others?

潜艇：小吴是一艘军用潜艇的船长。船上的爆炸导致潜艇失去了大部分氧气供应，并伤害了一名船员。受伤的船员无论怎样都将因伤势过重而死亡。同时剩余的氧气不足以让全体船员浮上水面。保存其他船员的唯一方法是射杀受伤的船员，这样剩下的船员就有足够的氧气生存。小吴为了保存其他船员的生命而杀死受致命伤的船员是否合适？

Euthanasia: Xiao Feng is the commander of a small group of soldiers, who are on their way back from completing a mission deep in enemy territory. One soldier steps into an enemy trap and is severely injured. The trap is connected to a radio device that has now alerted the enemy to the presence of Xiao Feng’s group. If the enemy discovers the injured soldier, they will torture and kill him/her. He/she pleads with Xiao Feng not to leave him/her behind. However, if Xiao Feng tries to take him/her along, the entire team will be captured. The only way to spare the injured soldier from torture is for Xiao Feng to shoot and kill him/her personally. Is it appropriate for Xiao Feng to shoot the soldier to prevent him/her from being tortured by the enemy?

安乐死：小冯是一小群士兵的长官，此时正在从敌人领土深处完成使命回来的路上，而一个士兵踩进了敌人设置的陷阱，受了重伤。这个陷阱连接到一个无线电装置上，这个装置现在已经提醒敌人注意到了小冯这群士兵的存在。如果敌人发现这个受伤的士兵，他们会折磨并杀死他（她）。他（她）恳求小冯不要丢下他（她）。但如果小冯试图带他（她）一起走，小冯的整个团队都会被抓住。唯一能让这个受伤的士兵不受折磨的办法就是小冯自己开枪杀死这个受伤的士兵。小冯为了防止这个士兵被敌人折磨而射杀他（她）是否合适？

### Appendix A.2

Forgot Lunch: Xiao Quan is hungry but forgot to bring lunch to work. There are no nearby restaurants, vending machines, or other food options. A colleague has a fridge stocked with snacks that could last about a week, but the colleague always refuses to share—if asked, they will definitely say no. Xiao Quan is considering secretly taking the colleague’s snacks without permission. How likely is Xiao Quan to take the colleague’s snacks?

忘记午餐：小全饿了，但忘了带午餐上班。附近没有餐馆、自动售货机或其他吃东西的地方。一位同事的冰箱里有大约一周的零食，但他（她）总是拒绝与人分享，如果有人向他（她）要吃的，他（她）肯定会拒绝。因此，小全在考虑是否自己偷偷拿他（她）的零食而不告诉别人。小全拿同事零食的可能性有多大？

Choking for Money: Xiao Li is waiting in a hospital lounge to visit a sick friend when seated beside a young person who reveals their father is critically ill, with doctors estimating a maximum one-week survival. The individual further explains their father has a substantial life insurance policy expiring at midnight: if the father dies before midnight, the young person will inherit a large sum, which they claim is “life-changing,” while their father’s extra days would yield no meaningful benefit. They offer Xiao Li ¥500,000 to smother the father with a pillow in his hospital room. Is it ethically justifiable for Xiao Li to kill the father for monetary gain?

为钱窒息：小李在医院休息室等着看望生病的朋友。坐在小李旁边的一个年轻人解释说他的父亲病得很重。医生认为他最多只能活一个星期。他（她）进一步解释说，他（她）的父亲有一份实质性的人寿保险，将在午夜到期。如果他（她）的父亲在午夜前去世，这个年轻人将得到一大笔钱。他（她）说这笔钱对他（她）来说意义重大，他（她）父亲再多活几天也不会有什么好处。他（她）给小李50万去他（她）父亲的房间用枕头闷死他（她）父亲。小李为了自己和这个年轻人的钱，杀了这个人的父亲合适吗？

The Architect: Xiao Wei is a young architect who is currently inspecting a construction site with his/her boss. The boss is a very despicable person who makes everyone around him, including Xiao Wei, miserable. Suddenly, Xiao Wei has the thought that if he/she pushes the boss off the building they are inspecting, the boss will fall to his death, and everyone will think it was an accident. Is it appropriate for Xiao Wei to push the boss off the building?

建筑师：小魏是一个年轻的建筑师，此时正在和他（她）的老板检查建筑工地。他（她）的老板是个非常卑鄙的小人，让周围的人都很痛苦，包括小魏。小魏突然想到，如果他（她）把老板从正在检查的大楼上推下来，老板就会摔死，每个人都会认为这是一场意外。小魏把老板从大楼上推下来的行为合适吗？

Standard Trolley: Xiao Yang is driving a runaway trolley that is rapidly approaching a fork in the tracks. On the left—extending track, there is a group of five railway workers. On the right track, there is only one railway worker. If Xiao Yang does nothing, the trolley will go to the left, resulting in the death of the five workers. The only way to prevent these workers from dying is to press a switch on the dashboard to make the trolley move to the right, causing the death of the single worker. Is it appropriate for Xiao Yang to press the switch to avoid the death of the five workers?

标准电车：小杨正驾驶着一辆失控的电车，快速接近铁轨上的一个岔路口。在向左延伸的铁轨上是一群五个铁路工人。右边的铁轨上只有一个铁路工人。如果小杨什么都不做，电车就会向左行驶，导致五名工人死亡。避免这些工人死亡的唯一方法是按下仪表板上的一个开关，使手推车向右移动，导致单个工人死亡。为了避免五个工人的死亡，小杨按下开关合适吗？

Extra Sweater: Xiao Zhang found that the package delivered by the postman contained an unworn sweater and some other purchased items. There was no charge for the sweater on the bill. Xiao Zhang tried on the sweater, and the color, style and fit were all very suitable! Xiao Zhang could easily return the sweater, as there were still some other things that didn’t fit and needed to be returned anyway. But the temptation to keep it was great. How likely is it that Xiao Zhang will keep the sweater without saying anything?

额外的毛衣：小张发现邮递员送来的包裹里有一件未购买的毛衣和其他一些已购买的物品。账单上并没有对毛衣收取任何费用。小张试穿了那件毛衣，颜色、款式和合身程度都非常吻合！小张可以很容易地把毛衣退掉，因为还有一些其他的东西不合适，无论如何都需要退掉。但保留它的诱惑还是很大。小张什么也不说，然后留着这件毛衣的可能性有多大？

Confidential Information: After a meeting, Xiao Dong’s new boss asked him/her to provide some confidential information about Xiao Dong’s former employer. How likely is it that Xiao Dong will provide this information to the new boss?

机密信息：在一次会议后，小东的新老板要求他（她）提供一些有关小东前雇主的机密信息。小东向新老板提供这些信息的可能性有多大？

Donation: One day, Xiaomi received a letter at home from a famous international aid organization. The letter requested Xiaomi to donate 200 yuan to their organization, explaining that the donation would enable the organization to provide medical services to some poor people in another part of the world. Is it appropriate for Xiaomi to refuse to donate to the organization in order to save money?

捐赠：有一天，小米在家里收到了一封来自著名国际援助组织的信件。信中要求小米向他们的组织捐款两百元。信中解释说，两百元的捐款将使该组织为世界上另一个地方的一些穷人提供医疗服务。小米为了省钱而不向该组织捐款是否合适？

Crying Baby: Enemy soldiers have taken over Xiao Qian’s village. They have been ordered to kill all remaining civilians. Xiao Qian and some villagers take refuge in the cellar of a large house. Xiao Qian hears the soldiers coming to search the house. At this moment, Xiao Qian’s baby starts crying loudly. Xiao Qian covers the child’s mouth to muffle the sound. If Xiao Qian removes her hand from the child’s mouth, the crying will attract the soldiers’ attention, and the soldiers will kill Xiao Qian, the child, and everyone hiding in the cellar. To save herself and the others, Xiao Qian must smother her own child. Is it appropriate for Xiao Qian to smother her child to save herself and the other villagers?

哭泣的婴儿：敌军士兵已经接管了小钱的村庄。他们奉命杀死所有剩余的平民。小钱和一些村民在一所大房子的地窖里寻求庇护。小钱听到士兵来搜查房子的声音。此时小钱的宝宝开始大声哭泣。小钱捂住孩子的嘴挡住声音。如果小钱把手从孩子嘴上拿开，孩子的哭声会引起士兵的注意，士兵会杀了小钱、孩子，还有躲在地窖里的所有人。为了拯救小钱和其他人，小钱必须把自己的孩子闷死。小钱为了拯救自己和其他村民而闷死自己的孩子合适吗？

Vaccine Test: A viral pandemic has spread across the Earth, causing millions of deaths. In the laboratory, Xiao Zheng has developed two substances. One of them is a vaccine, but Xiao Zheng does not know which one, while the other is a lethal poison. Once Xiao Zheng figures out which substance is the vaccine, it can be used to save millions of lives. At this moment, there are exactly two people in Xiao Zheng’s laboratory. The only way to identify the vaccine is to inject each of them with one of the two substances. One person will survive, and the other will die. Is it appropriate for Xiao Zheng to kill one of them in order to find a vaccine that can save millions of lives?

疫苗测试：一种病毒性流行病已经在地球上蔓延，造成了数百万人死亡。小郑在实验室里研制出了两种物质，其中一种是疫苗，但小郑不知道是哪一种，而另一种是致命的毒药。一旦小郑弄清楚哪种物质是疫苗，就可以用它来拯救数百万人的生命。此时刚好有两个人在小郑的实验室中，识别疫苗的唯一方法是给他们每人注射两种物质中的一种。一个人会活下来，另一个人会死。小郑为了找到一种能保存数百万人生命的疫苗，而杀死其中一个人合适吗？
